# Risk of self-harm in patients with hypochondriasis: a population-based cohort study

**DOI:** 10.3389/fpsyt.2025.1683539

**Published:** 2025-12-05

**Authors:** Wai Kwong Tang, Kelvin K. F. Tsoi, Terry Cheuk Fung Yip, Vivien Wei Jun Liew, Selina Kit Yi Chan

**Affiliations:** 1 Department of Psychiatry, The Chinese University of Hong Kong, Hong Kong, Hong Kong SAR, China; 2 Jockey Club School of Public Health and Primary Care, The Chinese University of Hong Kong, Hong Kong, Hong Kong SAR, China; 3 Stanley Ho Big Data Decision Analytics Research Centre, The Chinese University of Hong Kong, Hong Kong, Hong Kong SAR, China; 4 Department of Medicine and Therapeutics, The Chinese University of Hong Kong, Hong Kong, Hong Kong SAR, China

**Keywords:** hypochondriasis, self-harm, risk, depression, suicidal, cohort study

## Abstract

**Objective:**

This study aimed to ascertain whether individuals diagnosed with hypochondriasis (HYPO) exhibit a heightened risk of self-harming behaviors in comparison to those without the disorder.

**Methods:**

In this matched cohort study, we conducted a review of electronic health records of all patients admitted to Hong Kong public hospitals for any reason between January 1, 1993, and December 31, 2022. A HYPO cohort comprising 906 patients was identified, along with a comparison cohort of 906 matched subjects. All participants were followed until the occurrence of a self-harm diagnosis, death from other causes, or the end of 2023, whichever came first. The risk of self-harm from the onset of HYPO was estimated using Cox proportional hazards regression models and competing risk analysis.

**Results:**

Over the 30-year duration of the study, the number of individuals exhibiting self-harm behavior was 51 (5.6%) in the HYPO group and 49 (5.4%) in the comparison group. Both groups demonstrated comparable proportions of subjects engaging in self-harm (χ^2^ = 0.01, *p* = 0.927). The incidence rates of self-harm were 43.4 per 10,000 person-years in the HYPO group and 38.2 per 10,000 person-years in the comparison group. After adjustment, the hazard and subhazard ratio for self-harm in the HYPO group relative to the comparison group was 0.87 (95% confidence interval, 0.55–1.38) and 0.84 (95% CI, 0.53–1.34; p = 0.474), respectively.

**Conclusions:**

We did not find significant evidence for an elevated risk of self-harm associated with HYPO. Future research is warranted to replicate these findings and to more comprehensively identify the potential unique risk factors for self-harm in this patient population.

## Introduction

Hypochondriasis (HYPO), also known as illness anxiety disorder, is characterized by a persistent preoccupation with having one or more serious and progressive physical disorders (1). The preoccupation is accompanied by hypervigilance and a catastrophic interpretation of bodily signs. Abnormalities in the body, such as blemishes on the skin, protrusions and bumps, twitching of muscles, and pains and aches, are all given sinister significance and are regarded as harbingers of a serious illness (2), resulting in repetitive and excessive checking and reassurance-seeking behavior or maladaptive avoidance. The symptoms are clearly disproportionate and cause significant distress and impairment (1). Individuals with HYPO have high rates of medical consultations, typically leading to a chain of laboratory and other tests that are often unnecessary (3), and premature mortality (2). HYPO is thought to be severely underdiagnosed (4). However, as a symptom, health anxiety is highly prevalent in health care settings (5) and is associated with an elevated use of health care resources (6). The life prevalence rates of HYPO are 6% in the population and as high as 20% in hospital out-patients (5). HYPO affects men and women equally (7), and its onset typically occurs in early adulthood (7). In others, it develops as early as childhood or after an illness in adult life (5). HYPO is a chronic disease (7, 8). One study examined the outcomes of nearly 1,800 patients over 2 years. The findings showed that health-anxious patients remained unwell, with high scores throughout the 2 years, and their healthcare usage was 40–80% higher than the control group (4). In terms of risk factors, several life experiences have been proposed as precipitants of health anxiety, such as experiencing major life stress, a serious threat to one’s health, witnessing a loved one’s illness, and being exposed to health-threatening information (9). In terms of psychological mechanisms, it has been suggested that sub-optimal illness and health concepts, altered interoceptive modeling, biased illness-based predictions and attention, and aberrant prediction error learning are all relevant to HYPO (10). In terms of neurobiology, studies have shown the occurrence of increased amygdala activation, as well as increased activity in the rostral anterior cingulate cortex (11), right posterior parietal cortex, and nucleus accumbens (12), in response to body-symptom-related words in health-anxious individuals compared with healthy controls. In contrast, a recent study found no differences in amygdala activity or any brain activity between patients with health anxiety and healthy controls when exposed to health-related pictures (13). Treatment of HYPO commonly involves psychotherapy, including cognitive behavior therapy, stress management, mindfulness training, acceptance and commitment therapy, and antidepressants, namely serotonin-reuptake inhibitors (2).

HYPO is frequently associated with worse self-rated health; a more severe interruption of daily activities, such as household duties, self-care, and mobility; and more personal distress (7). Patients with HYPO often have comorbid major affective disorder (14). Given the negative consequences associated with HYPO and the elevated rates of co-occurring major depressive disorder, it is likely that the rate of suicide attempts is also elevated in this population.

Chronic health anxiety is thought to contribute to increased psychological distress (15, 16) which is recognized as a risk factor for self-injurious behavior in psychiatric populations (15, 17). Within the broader anxiety framework, health anxiety can be conceptualized as a condition driven by excessive fear and misinterpretation of benign bodily sensations, leading to recurrent reassurance-seeking and preoccupation with illness (18). These maladaptive cognitive-affective patterns often result in heightened emotional tension and reduced distress tolerance (15). Health-anxious individuals frequently become frustrated with unsuccessful reassurance-seeking, which can lead to intense emotional states such as despair and hopelessness (15). Following the cognitive model of suicidal behavior by (19), hopelessness may serve as a key mediator between somatoform-specific dysfunctional cognitive processes and suicidal ideation. Patients with somatoform disorders may develop profoundly dysfunctional beliefs about their unexplained physical symptoms, leading them to perceive their situation as hopeless and thereby increasing the likelihood of suicidal thoughts (19, 20).

Published data on suicide attempts or ideation in individuals with HYPO are very limited. In a cross-sectional study of 200 psychiatric inpatients, there was a non-significant trend towards patients with HYPO having a lower risk of suicidal attempt compared to those without HYPO (7% versus 14%) (21). In a Swedish nationwide cohort study, the unadjusted hazard ratio (HR) of suicidal death was 4.1, but the risk of suicidal death became nonsignificant after adjusting for depressive and anxiety disorders (22). A systematic review concluded that suicide attempts may be less frequent among individuals with HYPO than among those without (15). However, the above studies had a number of limitations. Subjects were recruited from inpatient settings (21); hence, the findings may not be generalizable to people with HYPO in the community or specific clinical practice situations. Suicidal death, rather than suicide attempts, were chosen as the outcome (22), and there are only a small number of relevant published papers (15). The purpose of the present study was to determine whether individuals with HYPO have an increased risk of self-harm behaviors relative to those without HYPO.

## Materials and methods

### Data source

In this matched cohort study, we utilized the Hong Kong Clinical Data Analysis and Reporting System (CDARS) to review electronic health records of all patients admitted for any reason to public hospitals in Hong Kong between January 1, 1993, and December 31, 2022. The CDARS is a validated and reliable electronic health record database managed by the Hospital Authority, the statutory body overseeing all public hospitals serving Hong Kong’s 7.7 million residents (23). It has been extensively used in prior epidemiological research due to its comprehensive coverage of inpatient, outpatient, and emergency services (23). Hong Kong’s current healthcare system delivers primary, secondary, and tertiary care through both public and private sectors (24), with Hospital Authority responsible for managing public healthcare services. Data from 2017 indicated that for approximately 80% of inpatient visits and 30% of outpatient visits in Hong Kong occur within the public system managed by the Hospital Authority (25). To protect patient privacy, CDARS encrypts patients’ personal information, providing researchers with anonymized identification numbers. The system’s diagnostic data have demonstrated high validity; for instance, a validation study found a positive predictive value of 96.8% for fracture diagnosis (26). This study received approval from the institutional review board of the Chinese University of Hong Kong (CREC 2020.707).

### HYPO cases

Cases were identified by selecting individuals who had received outpatient, emergency department, or inpatient care (general or psychiatric) with a diagnosis of HYPO during the study period. For each case, the date of the earliest HYPO diagnosis recorded in CDARS was designated as the commencement of the follow-up period. HYPO was identified using the International Classification of Diseases (ICD)-9 codes 300.70, 300.71, and 300.73. The diagnosis of hypochondriasis was made by medical doctors, both psychiatrists and non-psychiatrists can make the diagnosis. Individuals with prior self-harm or primary psychiatric disorders were not excluded.

### Comparison group without HYPO

To establish a comparable non-HYPO cohort, similar to the HYPO cases, we randomly chose individuals who had received outpatient, emergency department, or inpatient care (general or psychiatric) without prior history of HYPO during the study period, and matched them to the HYPO patients by sex and age at the time of recruitment. We assigned a random month and day, keeping the same index year as their matched HYPO counterparts. The matched admission date was used as the follow-up start date for all comparators.

### Covariates

For both the HYPO and comparison cohorts, data pertaining to ethnicity, residential district, and diagnosis of depression and bipolar disorders (ICD 9 code 296), anxiety disorder (ICD 9 code 300.0, 300.00-02, 300.09, 300.2-3), substance use (ICD 9 code 291-292, 303-305) and personality disorders (ICD 9 code 301.0-9) were extracted from the CDARS database. Comorbid physical diseases were measured by the Charlson Index (CCI) (27).

### Outcome measurement

All participants enrolled in this study were followed from the date of the earliest HYPO diagnosis recorded in CDARS or the matched admission date for comparators until the first occurrence of a self-harm diagnosis, death from any causes, or Dec 31, 2023, whichever occurred earliest. Given the tendency for self-harm events to be under-reported in hospital administrative datasets due to stigma and challenges in determining intent (28), we employed a broadened definition of self-harm beyond the standard ICD-9 classification. This definition accounts for all self-injurious behaviors, regardless of suicidal intent or whether the acts were intentional or unintentional. The ICD-9 codes utilized to identify self-harm were E950-59 (intentional self-harm) and E980-89 (self-harm with undetermined intent). Only self-harm that occurred after the diagnosis of hypochondriasis had been made were counted as event of interest.

### Statistical analysis

The number and proportion of individuals who engaged in self-harm were calculated for both the HYPO cases and their matched comparators. Differences in the proportions of self-harming individuals between the two groups were examined using the chi-square (χ^2^) test. Temporal patterns of self-harm following the earliest HYPO diagnosis recorded in CDARS were analyzed by plotting incidence curves using the Kaplan–Meier method, with statistical significance evaluated via the log-rank test. The incidence of self-harm was expressed as the number of self-harm per 10,000 person-years, calculated from the total follow-up time. Cox proportional hazards regression models were used to estimate the hazard ratio (HR) and the 95% confidence interval (CI) of the risk of self-harm since HYPO onset, accounting for time at risk and adjusting for relevant covariates, i.e., age, sex, ethnicity, residential district, and diagnosis of depression, bipolar disorders, anxiety disorders, personality disorders, substance use and CCI score. A competing risks regression was also performed, with death as a competing event in the estimation of self-harm risk. A sensitivity analysis was performed by limiting the event of interest to intentional self-harm (ICD E950–E959). Statistical analyses were conducted using STATA (StataCorp, College Station, TX, USA) with a *p* values threshold of < 0.05 considered indicative of statistical significance.

## Results

**Figure 1:**
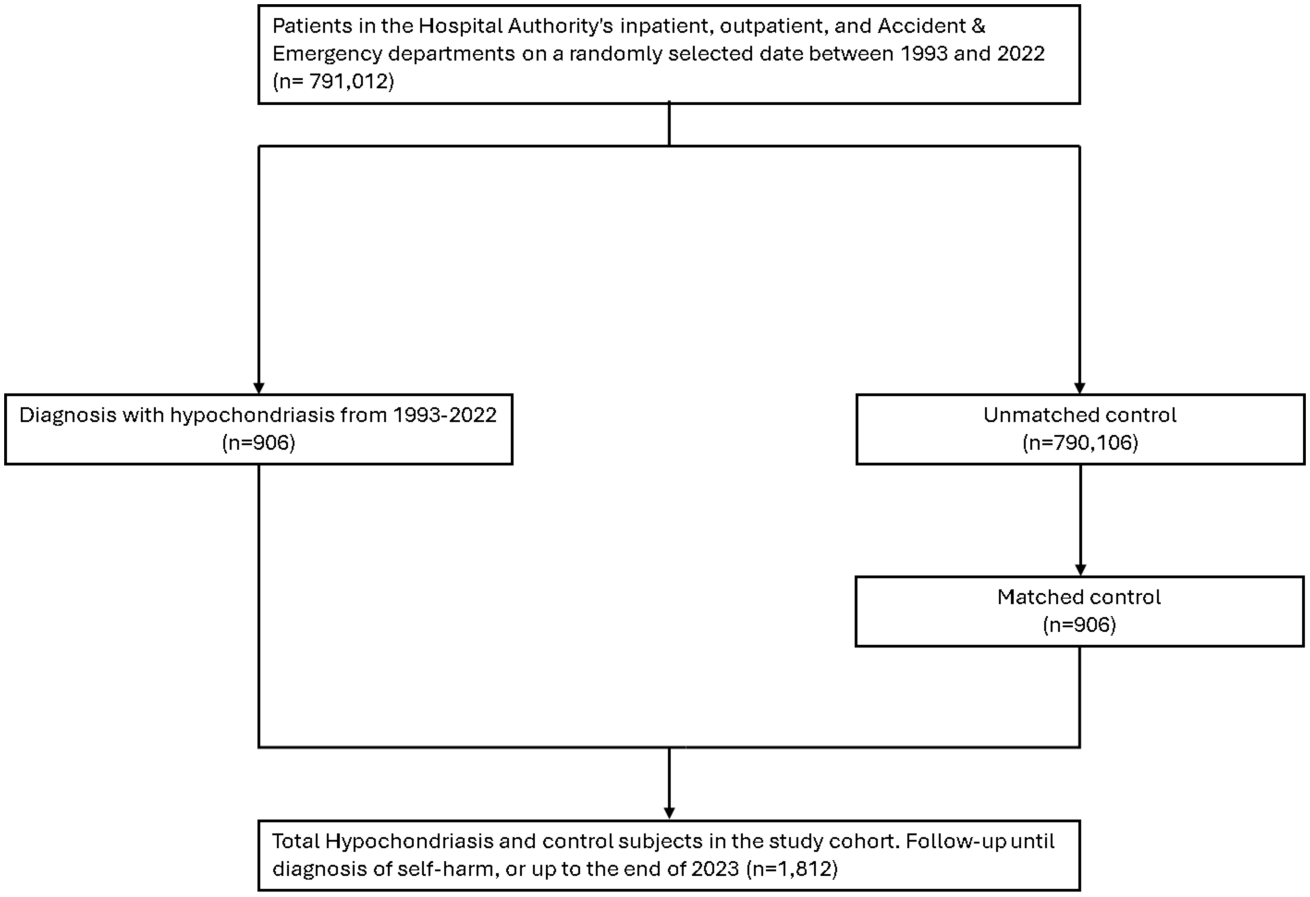
The flowchart of participant selection.

**Figure 2:**
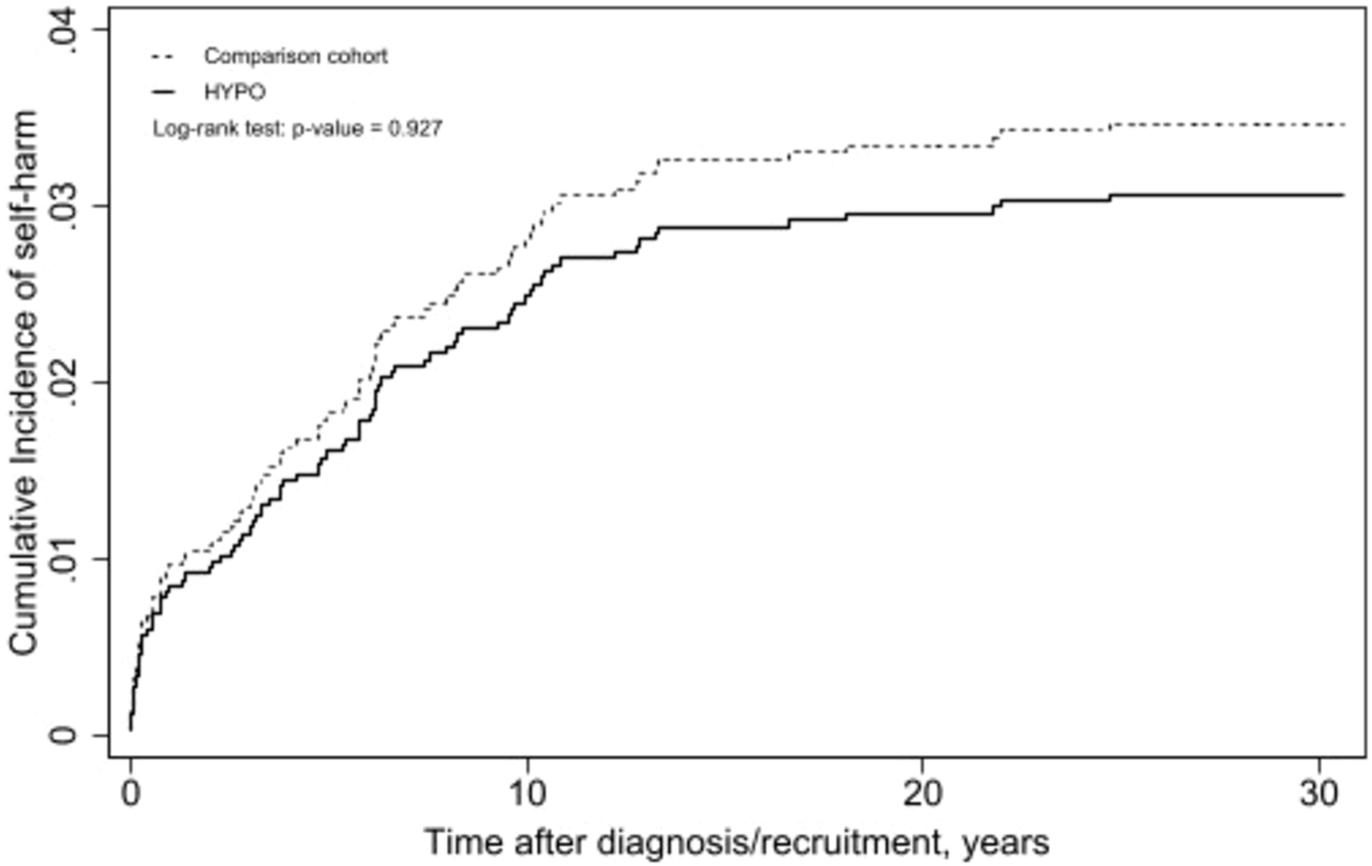
Kaplan-Meier analysis, adjusted for age, sex, ethnicity, residential district, history of depression, bipolar disorders, anxiety disorders, personality disorders, substance use, and CCI.

A cohort of 906 patients diagnosed with HYPO was selected, alongside a comparison cohort comprising consisting 906 matched subjects (**Figure 1**). The number of individuals exhibiting self-harm behavior was 51 (5.6%) in the HYPO group and 49 (5.4%) in the comparison group. The distributions of sex (50.3% female), ethnicity, age (48.5 ± 16.9 years), and residential district did not differ significantly between the HYPO and comparison cohorts. The HYPO group participants were more likely to have a history of depression/bipolar disorder, anxiety disorders, substance use, personality disorders and a lower CCI score (**Table 1**). Over the 30-year study period, the incidence rates of self-harm were 43.4 and 38.2 per 10,000 person-years in the HYPO and comparison groups, respectively. Kaplan–Meier analysis indicated that the incidences of self-harm were similar between the HYPO and comparison groups (log-rank *p* = 0.93; **Figure 2**). Compared to the comparison cohort, the unadjusted HR for self-harm in the HYPO group was 1.02 (95% CI, 0.68–1.54). After adjusting for age, sex, ethnicity, residential district, diagnoses of depression and bipolar disorders, anxiety disorders, substance use and personality disorders and CCI score, the HR in the HYPO group was 0.87 (95% CI, 0.55–1.38) relative to the comparison cohort. The mean number of self-harm occurrences was 5.5 ± 5.8 years in the HYPO group and 4.3 ± 4.8 years in the comparison group. The competing risk regression yielded a sub-distribution hazard ratio of 0.84 (95% CI, 0.53–1.34; p = 0.474) for self-harm in the HYPO group compared with the comparison group, indicating no significant difference in risk even after accounting for deaths. After restricting the event of interest to intentional self-harm only, the adjusted HR in the HYPO group was 0.82 (95% CI, 0.46–1.46) relative to the comparison cohort.

**Table 1:** Demographic and clinical characteristics in the study cohort.

Demographics/Clinical characteristics	Hypochondriasis (n=906)	Comparison cohort (n=906)	P value^a^
Gender			
Female	454 (50.31)	454 (50.31)	1.000
Male	452 (49.9)	452 (49.9)	
Age (mean ± SD)	48.5 ± 16.9	48.5 ± 16.9	1.000
Ethnicity^c^			
Chinese	851 (93.9)	859 (94.8)	0.325
Non-Chinese	55 (6.1)	46 (5.1)	
District of residence^d^			
Kowloon	407 (44.9)	315 (34.8)	0.356
New Territories	318 (35.1)	366 (40.4)	0.658
Hong Kong Island	181 (20.0)	219 (24.2)	0.137
History of depression/bipolar disorder	157 (17.3)	54 (6.0)	0.042
History of anxiety disorders	262 (28.9)	46 (5.1)	<0.001
History of personality disorders	44 (4.9)	27 (3.0)	0.040
History of substance use	58 (6.4)	85 (9.4)	0.019
CCI total score (mean ± SD, median (range))	1.5 ± 1.8	2.2 ± 2.3	<0.001
	1 (0-12)	2 (0-13)	
Time to self-harm (years) (mean ± SD)^b^	5.5 ± 5.8	4.3 ± 4.8	0.307

^a^Pearson chi-square test; ^b^T-test; ^c^1 subjects had no ethnicity data in comparison cohort, respectively, ^d^6 subject had no district of residence data in comparison cohort.

SD, Standard deviation; CCI, Charlson Comorbidity Index.

## Discussion

To the best of our knowledge, this study constitutes the first comprehensive examination of the risk of self-harm following a HYPO diagnosis in CDARS. No statistically significant difference in self-harm incidence was detected between the patients with HYPO and their matched comparators, even after adjusting for coexisting mood disorders.

In this study, the risk of self-harm among patients with HYPO was comparable to that observed in the matched comparator group. In a previous cross-sectional study, there was a non-significant trend that patients with HYPO had a lower risk of suicide attempts than those without HYPO (7% versus 14%) (21). In a cohort study, the unadjusted HR of suicidal death was 4.1, but the risk of suicidal death became nonsignificant after adjusting for depressive and anxiety disorders (22). In contrast, studies on somatoform and somatization disorders have reported an elevated risk of suicide attempts in affected individuals. In a study of psychiatric outpatients in Norway, patients with somatization disorder were more likely to report a past suicidal attempt (odds ratio [OR] = 4.5) (29). In another study of adolescents and young adults in Germany, baseline somatoform disorder was predictive of reports of suicide attempts at final assessment (OR = 5.6) (29). In a third study conducted in Korea, somatoform disorders were associated with a high risk of suicide attempt (OR = 6) (30). Possible explanations for the negative results in the present study include the under-reporting of self-harm in the HYPO group and an elevated risk of self-harm in the comparison group. There were also methodological differences between these studies and our study, namely, the cross-sectional design (30), involvement of psychiatric outpatients (31) or inpatients (21) or adolescents and young adults (29), retrospective diagnosis of somatoform disorder (29), reliance on self-report of suicide attempts (30), and use of suicidal death as the outcome (22).

In the present study, 5.6% of patients had inflicted self-harm, which is similar to the rate of suicide attempts (7%) recorded in a cross-sectional study (21). In a nationwide cohort study, the rate of suicidal death was 0.7% (22). In contrast, studies on somatoform and somatization disorders have reported higher rates of suicide attempts in these individuals (13% to 51%) (20, 31–34). Possible explanations for these differences could be the nature of the sample and method of collecting self-harm data. In the present study, the sample was population-based rather than a sample of convenience. In addition, all self-harm episodes were ascertained by electronic medical record instead of self-reporting.

The absence of a significant association between HYPO and self-harm risk provided important theoretical and clinical insight. Conceptually, these findings suggested that the cognitive-affective mechanisms underlying HYPO, such as excessive health-related rumination, reassurance seeking, and fear of having a disease, may operate within an anxiety framework distinct from the affective pathways typically linked to self-directed harm (18–20, 35). Although individuals with HYPO often experience heightened distress and perceived helplessness (15), these experiences may not involve the self-critical or punitive cognitions, which is characteristic of depressive pathology that tend to drive self-harm behavior (35, 36). This pattern suggests that HYPO alone may not elevate self-harm risk, but risk could arise when HYPO coexists with other vulnerabilities such as comorbid depression or emotional dysregulation (15, 22). Notably, Torres et al. (15) reported that somatic symptom and related disorders may confer independent suicidality risk even after controlling for affective comorbidities, indicating potential subgroups with distinct mechanisms of distress. Together, these findings suggested that the relationship between HYPO and self-harm is complex and likely contingent upon comorbid psychopathology and underlying cognitive-affective dynamics. Clinically, these findings suggested that self-harm risk assessments should consider not only health anxiety symptoms but also comorbid affective or personality factors that may more directly contribute to self-injurious behaviors. Despite the absence of an overall elevated risk, clinicians should maintain vigilance for suicide risk among HYPO patients, particularly those with comorbid depression or affective disorders. For HYPO patients with no comorbid affective disorders, clinicians may prioritize interventions that target health-related distress, such as cognitive-behavioral therapy (CBT) for health anxiety (18), without assuming an inherent elevation in self-harm risk. Given that depressive and other psychiatric comorbidities can coexist with hypochondriacal features, future research should examine how these conditions moderate the link between health anxiety and self-harm. Large-scale longitudinal and multicenter studies are warranted to replicate these findings and to identify contextual factors, such as healthcare utilization, illness beliefs, and coping strategies, that may influence self-harm vulnerability in somatic symptom-related disorders.

This study has several limitations. The sample was derived from medical records within the CDARS, excluding data from private hospitals, private clinics, and general outpatient clinic services. Consequently, the comparison cohort, drawn from hospital and outpatient clinic databases, may have had a higher baseline risk of self-harm than the general population, potential resulting in an underestimation of HRs. Nonetheless, the study design incorporated a suitable comparison group matched for age and sex to mitigate confounding. In addition, we did not have access to data on socioeconomic status.

We employed an inclusive definition of self-harm encompassing deliberate self-injurious behaviors with suicidal intent; non-suicidal self-injurious; as well as both intentional and unintentional self-injurious acts. A sensitivity analysis from a study examining the risk of self-harm among psychiatric patients in Hong Kong indicated that excluding cases of undetermined self-harm (ICD-9 codes E980–89) did not substantially alter the findings (37). Additionally, our dataset lacked information regarding patient interventions and treatments, which may have impacted the study results. Notably, the CDARS records only suicidal actions, whereas the prevalence of suicidal ideation is likely higher and may be under-reported in clinical settings.

We aimed to investigate the association between the onset of HYPO and subsequent self-harm behaviors. However, as clinical records prior to 1993 were unavailable, we could not confirm whether the earliest HYPO diagnosis recorded in CDARS corresponded to the true onset of HYPO. Additionally, data on self-harm were limited to medical records, which may be subject to under-reporting due to the nature of administrative data collection.

Although our 30-year cohort provided valuable long-term insight, the relatively small number of self-harm events resulted in wide confidence intervals and limited statistical power, meaning the analysis may not reliably detect a modest association between groups. Therefore, the absence of statistical significance should not be interpreted as confirmation of equivalence, wherein the possibility of a type II error remains a concern.

Finally, self-harm is a complex behavior affected by several factors, e.g., demographic, social, economic, cultural, psychological, and environmental factors (38). However, due to limitations of the data source, we were unable to incorporate these risk factors in our analysis. Ideally, comprehensive information on sociodemographic variables such as marital status, employment status, and education level, along with potential confounders including physical comorbidities, smoking, and alcohol use, should be available and accounted for through appropriate adjustments.

## Conclusions

We did not find significant evidence for an elevated risk of self-harm associated with HYPO. However, given the statistical uncertainty, this should not be taken as definitive proof of no association. Clinical vigilance remains important especially for HYPO patients with comorbid affective disorders. Future research is warranted to replicate these findings and to more comprehensively identify the potential unique risk factors for self-harm among this patient population.

## Data Availability

The data analyzed in this study is subject to the following licenses/restrictions: The data used in this study were obtained from the Clinical Data Analysis and Reporting System (CDARS) managed by the Hospital Authority (HA) in Hong Kong. Due to privacy and legal restrictions, data access is strictly controlled and limited to anonymised datasets, subject to HA approval. Data request must be made through the HA’s official channels. Requests to access these datasets should be directed to Hospital Authority, https://www3.ha.org.hk/data/Provision/ApplicationProcedure.
